# MSCs enhances the protective effects of valsartan on attenuating the doxorubicin-induced myocardial injury via AngII/NOX/ROS/MAPK signaling pathway

**DOI:** 10.18632/aging.203569

**Published:** 2021-09-29

**Authors:** Dong Cheng, Wencheng Tu, Libo Chen, Haoren Wang, Qinfu Wang, Hainiang Liu, Ning Zhu, Weiyi Fang, Qin Yu

**Affiliations:** 1 Department of Cardiology, Affiliated Zhongshan Hospital of Dalian University, Dalian 116001, Liaoning, China; 2 Medical College, Dalian University, Dalian 116622, Liaoning, China; 3 Department of Cardiology, Jingmen No.1 People’s Hospital, Jingmen 448000, Hubei, China; 4 Department of Cardiology, People’s Hospital of Jilin City, Jilin 132000, Jilin, China; 5 Central Laboratory, Affiliated Zhongshan Hospital of Dalian University, Dalian 116001, Liaoning, China; 6 Life Engineering College, Dalian University, Dalian 116622, Liaoning, China; 7 Department of Cardiology, The Second Affiliated Hospital of Dalian Medical University, Dalian 116023, Liaoning, China; 8 Department of Cardiology, Shanghai Chest Hospital, Changning 200030, Shanghai, China

**Keywords:** mesenchymal stem cells, doxorubicin, MAPK signaling pathway, valsartan, ROS

## Abstract

Objective: To verify if AngII/NOX/ROS/MAPK signaling pathway is involved in Doxorubicin (DOX)-induced myocardial injury and if mesenchymal stem cells (MSCs) could enhance the protective effects of valsartan (Val) on attenuating DOX-induced injury *in vitro*.

Methods: Reactive oxygen species (ROS) formation and the protein expression of AT1R, NOX2, NOX4, caspase-3, caspase-9 and MAPK signaling were assessed in H9c2 cardiomyocytes exposed to DOX for 24 h in the absence or presence of Val, NADPH oxidase inhibitor DPI or knockdown and overexpression of NADPH oxidase subunit: NOX2 and NOX4, co-culture with MSCs, respectively. Finally, MTT assay was used to determine the cell viability of H9c2 cells, MDA-MB-231 breast cancer cells and A549 pulmonary cancer cells under Val, DOX and Val+ DOX treatments.

Results: DOX increased ROS formation and upregulated proteins expression of AT1R, NOX2, NOX4, caspase-3, caspase-9 and MAPK signaling including p-p38, p-JNK, p-ERK in H9c2 cells. These effects could be attenuated by Val, DPI, NOX2 siRNA and NOX4 siRNA. Meanwhile, overexpression of NOX2 and NOX4 could significantly increase DOX-induced ROS formation and further upregulate apoptotic protein expressions and protein expressions of MAPK signaling. MSCs on top of Val further enhanced the protective effects of Val on reducing the DOX-induced ROS formation and downregulating the expression of apoptotic proteins and MAPK signaling as compared with Val alone in DOX-treated H9c2 cells. Simultaneous Val and DOX treatment did not affect cell viability of DOX-treated MDA-MB-231 breast cancer cells or A549 pulmonary cancer cells but significantly improved cell viability of DOX-treated H9c2 cardiomyocytes.

Conclusions: AT_1_R/NOX/ROS/MAPK signaling pathway is involved in DOX-induced cardiotoxicity. Val treatment significantly attenuated DOX-induced cardiotoxicity, without affecting the anti-tumor effect of DOX. MSCs enhance the protective effects of Val on reducing the DOX-induced toxicity in H9c2 cells.

## INTRODUCTION

As an anthracycline chemotherapeutic drug, Doxorubicin (DOX) is worldwide used in the treatment of solid tumors, leukemias, lymphomas and breast cancer [1]. Unfortunately, severe, irreversible and dose-dependent cardiac toxicity limits DOX in clinical application [2] The mortality rate of DOX-induced cardiomyopathy is as high as 30% to 50%. There is no absolute safe dose due to individual differences and high risk of minors [3]. Therefore, the prevention of DOX-induced cardiotoxicity is of clinical importance.

Based on previous reports [4, 5], dexrazoxane serves as the only drug recommended by guideline to prevent DOX-induced cardiotoxicity, which can reduce its occurrence by 50% [6]. However, the clinical applications of dexrazoxane is limited by its strict indications and high cost [7–9]. Numerous strategies, including adjustment of the medication regimen, reduction of cumulative dose and the peak plasma concentration and switching to the liposome dosage form, are somewhat effective, but the efficacy of the strategies used in reducing DOX-induced cardiotoxicity still remains unsatisfactory [10].

Previous study found that angiotensin II (AngII) blocker was effective in attenuating DOX-induced cardiotoxicity [11] and our previous study also showed that repeated intravenous injection of mesenchymal stem cells (MSCs) could significantly ameliorate DOX-induced myocardial damage in rats [12]. It is known that the DOX-induced excessive ROS generation is a major mechanism of DOX-induced myocardial damage [5], which could also cause activation of the renin-angiotensin-aldosterone system (RASS) [13], activation of the MAPK pathway, leading to apoptosis of cardiomyocytes [14, 15]. Besides, NADPH oxidase (NOX) signaling is also activated in DOX-induced cardiac insult model [16]. Thus, NOX/ROS/MAPK signaling pathway plays a central role in the pathogenesis of DOX-induced cardiotoxicity. In the present study, we explored the impact of DOX on NOX/ROS/MAPK signaling pathway *in vitro* using H9c2 cells and observed the impact of AngII receptor blocker valsartan (Val) in the presence or absence of MSCs on attenuating DOX-induced toxicity in H9c2 cells and related mechanisms via NOX/ROS/MAPK signaling pathway.

## MATERIALS AND METHODS

### Chemicals, reagents and antibodies

DOX was purchased from Zhejiang Hisun Pharmaceutica Co, Ltd. (Taizhou, China); Val and Diphenyleneiodonium chloride (DPI) were purchased from Meilunbio Inc (Dalian, China); MTT reagent and Apoptosis Detection Kit were purchased from Sigma-Aldrich Inc (WI, USA); NOX2 siRNAs, NOX4 siRNAs, negative siRNA, NOX2 plasmid, NOX4 plasmid and empty vector were constructed by GenePharma Inc (Shanghai, China); Lipofectamine 2000 and Protein marker (PageRuler™) were purchased from Invitrogen (CA, USA); Trizol reagents and PCR kit were purchased from Takara Bio Inc (Dalian, China); The primer sequence of NOX2, NOX4 and GAPDH were synthesized by ShineGene Inc (Shanghai, China); Primary monoclonal antibodies for Angiotensin II Type 1 Receptor (AT1R) (ab124734), NOX2/gp91phox (ab129068), NOX4 (ab79971), caspase-3 (ab184787), caspase-9 (ab184786), p-p38 (ab4822), p38 (ab170099), p-JNK (ab124956), JNK (ab179461), p-ERK (ab201015), ERK (ab184699), β-actin (ab8227) and secondary antibodies (ab6721) were purchased from Abcam (Cambridge, UK).

### Preparation of cells

H9c2, human breast cancer MDA-MB-231 and human pulmonary carcinoma A549 cell lines were purchased from China Center for Type Culture Collection (CCTCC), they were cultured in Dulbecco’s minimum essential medium (DMEM) with 10% Fetal Bovine Serum (FBS), AusGeneX (Brisbane, AUS), penicillin (100 U/mL), and streptomycin (100 μg/mL) and incubated in 95 % air and 5 % CO_2_ at 37° C temperatures.

Preparation of MSCs. Bone marrow derived MSCs were obtained and identified as describe previously [12].

Co-cultures of MSCs and H9c2 cells. To detect the effect of MSCs combined with Val on DOX-treated cardiomyocytes, H9c2 and MSCs were co-cultured in the Transwell^®^ system (Corning Inc. (NY, USA)) for 24 h, pretreated with Val for 1 h, then treated with DOX for 24h. Transwell membrane plate were used to avoid the direct contact of MSCs with H9c2 cells, but permit the free cell culture medium exchange between the two layers.

### Cell viability analysis

MTT assay was used to determine the cell viability. 3×10^4^ cells/well H9c2 cells were seeded on 96 well plates and 4.5×10^3^ cells/well MDA-MB-231 cells and A549 cells were seeded on 96 well plates overnight at 5% CO_2_, 37° C incubator. Val was dissolved in anhydrous ethanol (the final working concentration of anhydrous ethanol should not exceed 0.01%) and DOX was dissolved in deionized water. H9c2 cells, MDA-MB-231 cells or A549 cells pre-treated with Val (5 μM) for 1 h followed by DOX (1 μM) for 24 h. Subsequently, 20 μL MTT (5 mg/mL) was added to each well, 4 hours later, cell viability was detected through measuring the absorbance at 490 nm by a microplate reader.

### Detection of intracellular ROS

Intracellular ROS was measured by monitoring the DCF fluorescence. Briefly, 5×10^5^ H9c2 cells were seeded in 6-well plate overnight. Then pre-treated with Val (5 μM) for 1 h followed by DOX (1 μM) for 0 h, 3 h, 12 h and 18 h. H9c2 and MSCs were co-cultured in the transwell system for 24 h, then the co-culture system was pretreated with Val for 1 h. After treatment with DOX for 12h, the H9c2 cells were dyed with 10 μM DCF-DA for 30 minutes, then DCF fluorescence was detected under fluorescence microscope, BD FACSDiva software was used to analyze the intracellular ROS semi-quantitatively.

### Analysis of cardiomyocyte apoptosis

H9c2 cells was pre-treated with Val (5 μM) for 1 h followed by DOX (1 μM) for 24 h in 6-well plates, then suspended in 195 μL binding buffer and mixed with 5 μL Annexin V-FITC and 10 μL PI solutions. After placed in the dark for 15 min, apoptosis rate of H9c2 cells was detected by flow cytometer.

### Small interfering RNA (siRNA) transfection of NOX2 and NOX4

According to the manufacturer's protocol and literature [17], the siRNA targeting NOX2 siRNA, NOX4 siRNA and the negative siRNA were transiently transfected into cells. The sequences of siRNAs are shown in Table 1.

**Table 1:** siRNA sequences.

**siRNA**	**Sense (5′-3′)**	**Anti-sense (5′-3′)**
negative	GCGACGAUCUGCCUAAGAUdTdT	AUCUUAGGCAGAUCGUCGCdTdT
Nox2-rat-587	CCAUUCGGAGGUCUUACUUTT	AAGUAAGACCUCCGAAUGGTT
Nox2-rat-663	CCAUGGAGCUGAACGAAUUTT	AAUUCGUUCAGCUCCAUGGTT
Nox2-rat-1412	CCAACUUCCUCAGCUACAATT	UUGUAGCUGAGGAAGUUGGTT
Nox4-rat-576	GCUUCUACCUAUGCAAUAATT	UUAUUGCAUAGGUAGAAGCTT
Nox4-rat-912	GGACCUUUGUGCCUAUACUTT	AGUAUAGGCACAAAGGUCCTT
Nox4-rat-1048	GACCUGGCCAGUAUAUUAUTT	AUAAUAUACUGGCCAGGUCTT

### Overexpression of NOX2 and NOX4 with plasmid

Restriction enzyme sites (XhoI and KpnI) was used to edit the vector that cloned NOX2 and NOX4 genes into pEX-4. The primer sequences of plasmids are shown in Table 2. The detailed protocol of PEX-4-NOX2/NOX4 or empty vector transfected into H9c2 cells via Lipofectamine 2000 as described previously [18].

**Table 2:** Primer sequences of plasmids.

**Plasmid**	**Forward primer (5′-3′) XhoI**	**Reverse primer (5′-3′) KpnI**
NOX2	GCGCTACCGGACTCAGATCTCGAGGCCACC ATGGGGAACTGGGCTGTGAATGAGGGACTC	ACTTCCTCTGCCCTCGGTACCGAAGTTTTC CTTGTTGAAGATGAAGTGGACTCCACGTGG
NOX4	GCTACCGGACTCAGATCTCGAGGCCACCAT GGCGCTGTCCTGGAGGAGCTGGCTGGCCAA	ACTTCCTCTGCCCTCGGTACCGCTGAAAGA TTCTTTATTGTATTCAAATTTTGTCCCATA

### Real-time PCR

The total RNA of H9c2 cells was extracted and quantified after gene silencing and overexpression. The primers for real-time PCR are shown in Table 3. Real-time PCR was performed as described previously [19].

**Table 3:** Primer sequences of qPCR.

**Gene**	**Forward primer (5′-3′)**	**Reverse primer (5′-3′)**
NOX2	CCATTCACACCATTGCACATC	CGAGTCACAGCCACATACAG
NOX4	GAACCCAAGTTCCAAGCTCA	GCACAAAGGTCCAGAAATCC
GAPDH	GGTGCTGAGTATGTCGTGGAGT	CACAGTCTTCTGAGTGGCAGTG

### Western blot analyses

H9c2 cells proteins under various treatments were collected by adding cell lysis buffer. The homogenate was centrifuged and quantified, followed by equivalent proteins separation, blocked and incubated overnight with AT_1_R, NOX2/gp91phox, NOX4, caspase-3, caspase-9, p-p38, p38, p-JNK, JNK, p-ERK, ERK and β-Actin antibodies at 4° C under gentle agitation, then incubated with secondary antibody. The protein bands were exposed by a chemiluminescent agent. The quantify protein expressions was analyzed with ImageJ software. All the study protocols were approved by the Institutional Animal Research and Ethics Committee of Dalian University, China.

### Statistical analysis

Data (mean ± SE) were analyzed by one-way ANOVA followed by Bonferroni’s post hoc comparisons with the SPSS statistical program (SPSS software Version 22.0). A p value <0.05 was considered as statistically significant.

## RESULTS

### Cell viability determination by MTT assay

**Figure 1:**
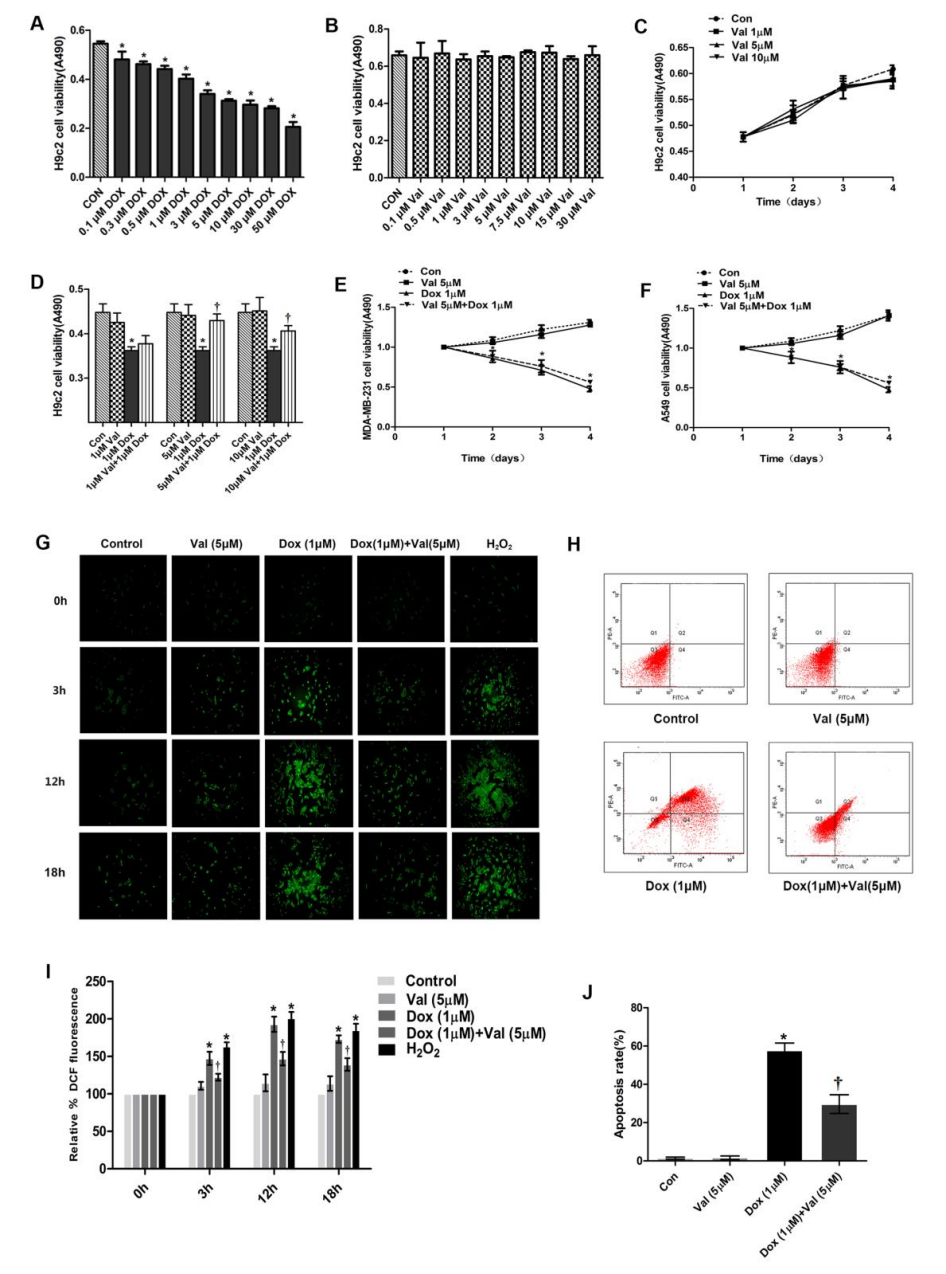
**The cell viability determination and effect of Val pre-treatment followed by DOX-exposure on ROS level and apoptosis rate in H9c2 cells.** (**A**) Effect of DOX on H9c2 cells viability for 24 h. (**B**) Effect of Val on H9c2 cells viability for 24 h. (**C**) Effect of Val at three concentrations on H9c2 cells over time. (**D**) Viability of DOX-induced H9c2 cells followed as three of Val concentrations pre-treatment. (**E**, **F**) Effect of Val and DOX on tumor cells viability. (**G**) Effect of Val pre-treatment on the ROS level of DOX-treated H9c2 cells at different time points. (**H**) Effect of Val on apoptosis of DOX-treated H9c2 cells. (**I**) Microscopic analysis of Val and DOX-treatment on ROS levels by DCF Fluorescence. (**J**) The bar graph of the quantitative analysis of apoptosis rate of H9c2 cells. *P<0.05 vs. Control; †P<0.05 vs. DOX. DOX, doxorubicin; Val, valsartan.

H9c2 cells were treated with DOX at different final concentration (0.1, 0.3, 0.5, 1, 3, 5, 10, 30, 50 μM) for 24 h. The results showed that DOX induced a dose-dependent reduction in cell viability (Figure 1A). 1 μM DOX-treatment decreased cell viability to 55%, this concentration is similar to the dose used in the clinical DOX-chemotherapy. Hence, further experiments were carried with 1 μM DOX; H9c2 cells were incubated with different final concentration of Val (0.1, 0.5, 1, 3, 5, 7.5, 10, 15, 30 μM) for 24 h, cell viability post Val treatment at various concentrations was similar to the control group (Figure 1B). Three Val concentrations (1, 5 and 10 μM) were used, the results showed that Val did not affect the growth of H9c2 cells over time (Figure 1C). The Val concentration required to protect against DOX-induced cytotoxicity was calculated by performing a dose-response study in the presence of 1, 5 and 10 μmol/L Val (Figure 1D). The cytotoxic effects of DOX were significantly attenuated by 5 and 10 μmol/L Val pretreatment. Based on these results, minimum effective concentration (5 μM) Val was chosen for further studies. MDA-MB-231 cells and A549 cells were pre-treated with 5 μM Val for 1 h followed by treatment with 1 μM DOX for 24 h. Val did not affect the function of DOX on tumor cells (Figure 1E, 1F).

### Detection of intracellular ROS in H9c2 cells

We detected the ROS generation in H9c2 cells in a time course experiment (0 h, 3 h, 12 h and 18 h) to determine ROS generation post DOX stimulation. DOX-induced ROS generation started from 0 h, peaked at 12 h, and then began to decrease (Figure 1G). The expression of ROS in DOX group was significantly higher than that in the control group, which could be significantly reduced by Val (Figure 1I).

### Flow cytometric analyses of cardiomyocyte apoptosis

The apoptosis rate in the DOX group was significantly higher than that in the control group, which could be significantly reduced by Val (Figure 1H, 1J).

### Val and DPI reduced expression of proteins and ROS level in DOX-treated H9c2 cells

**Figure 2:**
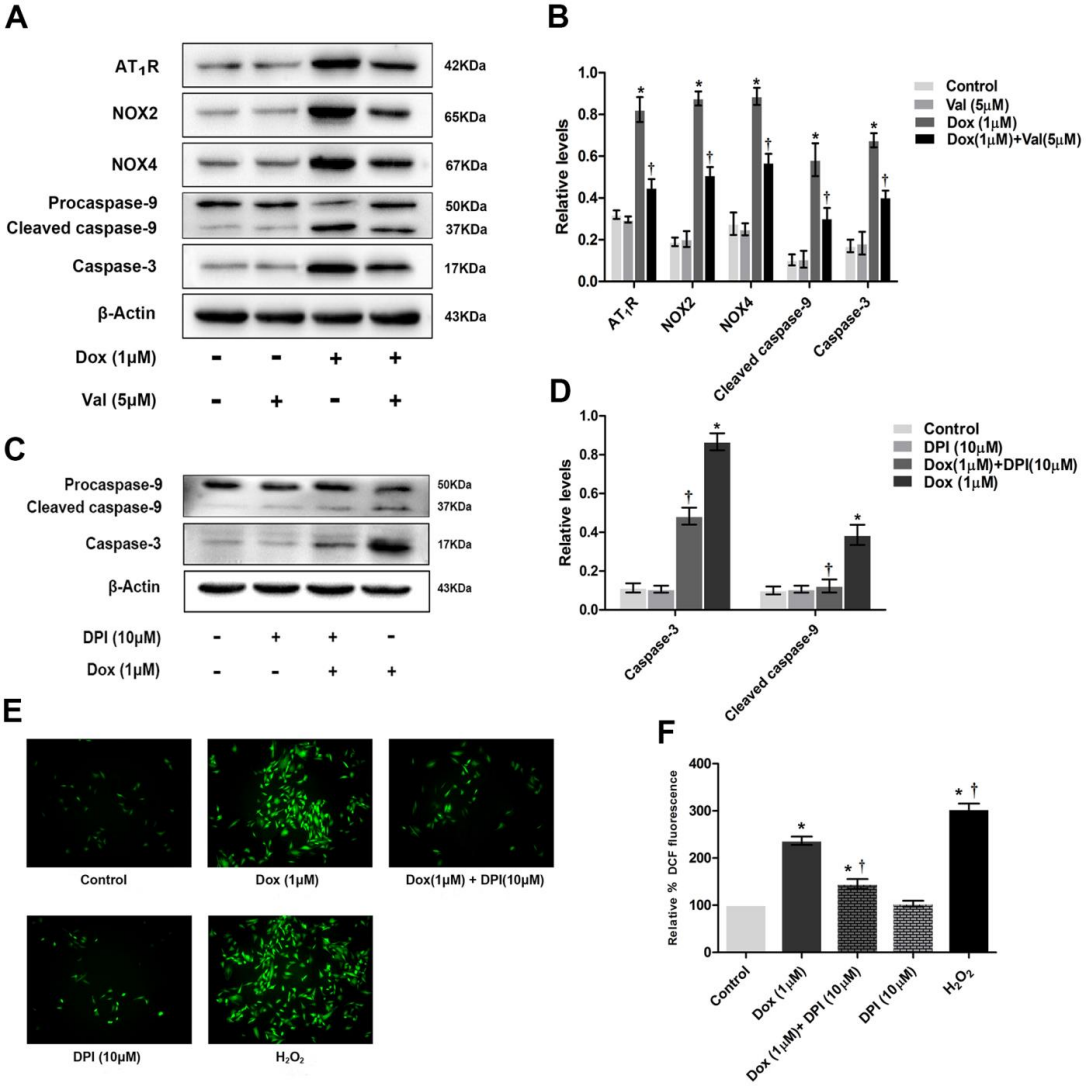
**The effect of Val pre-treatment and DPI followed by DOX-exposure on expression of proteins and ROS level in H9c2 cells.** (**A**) Western blot analysis of Val and DOX-treatment on AT1R, NOX2, NOX4, caspase-3, cleaved caspase-9 protein levels. (**B**) Densitometry analysis of the protein bands of AT1R, NOX2, NOX4, caspase-3, cleaved caspase-9 proteins. (**C**) Western blot analysis of DPI and DOX-treatment on caspase-3, cleaved caspase-9 protein levels. (**D**) Densitometry analysis of the protein bands of caspase-3, cleaved caspase-9 proteins. (**E**) Effect of DPI and DOX on ROS levels at 12 h time point. (**F**) Microscopic analysis of DPI and DOX-treatment on ROS levels by DCF Fluorescence. *P<0.05 vs. Control; †P<0.05 vs. Dox. Dox, doxorubicin; Val, valsartan.

The expression of AT_1_R, NOX2 and NOX4 in the DOX group was significantly higher than that in the control group. The expression of AT_1_R, NOX2 and NOX4 was significantly lower in DOX+Val group than that in DOX group (Figure 2A, 2B). The expression of caspase-3 and cleaved caspase-9 in DOX group was significantly higher than that in the control group. The expression of caspase-3 and cleaved caspase-9 was significantly lower in DOX+Val group than that in DOX group (Figure 2A, 2B). Taken together, Val can downregulate the expression of AT_1_R, NOX2, NOX4, caspase-3, cleaved caspase-9 and the content of ROS in H9c2 cells stimulated by DOX. DPI is an inhibitor of NADPH oxidase, which inhibits both NADPH subunits NOX2 and NOX4 [20]. The expression of caspase-3 and cleaved caspase-9 in DOX+DPI group was significantly lower than that in DOX group (Figure 2C, 2D). The expression of ROS in DOX+DPI group was significantly lower than that in DOX group (Figure 2E, 2F). These results indicate that DPI can reduce the expression of caspase-3, cleaved caspase-9 and the content of ROS in H9c2 cells stimulated by DOX. This result indicates that NADPH activation plays a crucial role in DOX-induced cytological toxicity.

### Knockdown of NOX2 and NOX4 reduced ROS generation, caspase-3, and cleaved caspase-9 expression in DOX-treated H9c2 cells

**Figure 3:**
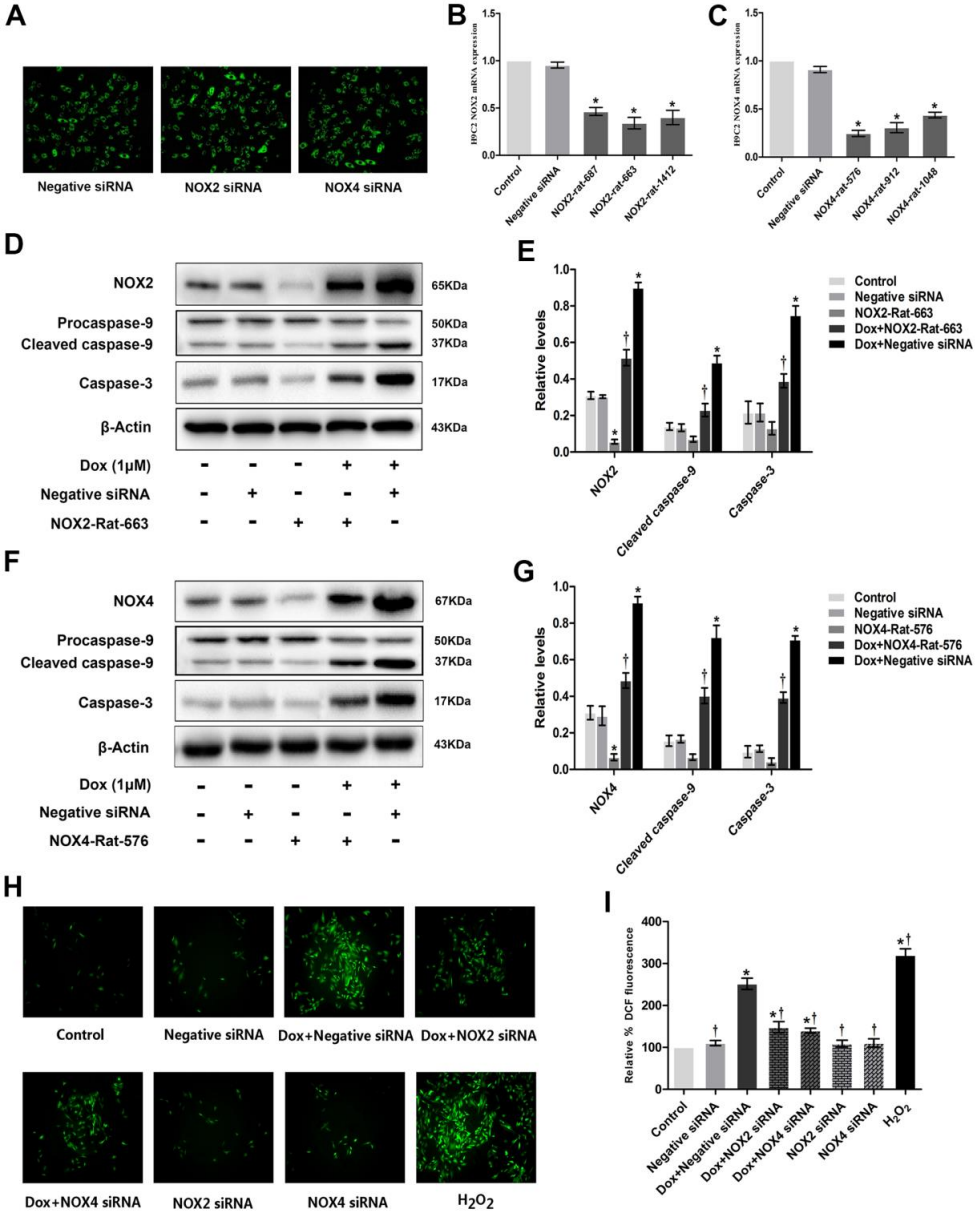
**Knockdown of NOX2 and NOX4 expression could reduce DOX-induced ROS production and expression of apoptotic proteins in H9c2 cells.** (**A**) Fluorescence microscope observation of H9c2 cells after transfecting with Negative siRNA, NOX2 siRNA and NOX4 siRNA (×100). (**B**) Three distinct NOX2 siRNAs and one negative siRNA were designed, and Nox2-rat-663 was selected as the best interference fragment by PCR. (**C**) Three distinct NOX4 siRNAs and one negative siRNA were designed, and Nox4-rat-576 was selected as the best interference fragment by PCR. (**D**) Western blot analysis of NOX2 siRNA and DOX-treatment on NOX2, caspase-3, cleaved caspase-9 protein levels. (**E**) Densitometry analysis of the protein bands of NOX2, caspase-3, cleaved caspase-9 proteins. (**F**) Western blot analysis of NOX4 siRNA and DOX-treatment on NOX4, caspase-3, cleaved caspase-9 protein levels. (**G**) Densitometry analysis of the protein bands of NOX4, caspase-3, cleaved caspase-9 proteins. (**H**) Effect of NOX2 siRNA, NOX4 siRNA and DOX-treatment on ROS levels at 12 h time point in H9c2 cells. (**I**) Microscopic analysis of NOX2 siRNA, NOX4 siRNA and DOX-treatment on ROS levels by DCF Fluorescence. *P<0.05 vs. Control; †P<0.05 vs. Dox. DOX, doxorubicin; siRNA, small interfering RNA.

H9c2 cells were transfected with Negative siRNA, siRNA-NOX2 and siRNA-NOX4, respectively. The results show that NOX siRNA has a good transfection effect (Figure 3A) and knockdown efficiency (Figure 3B, 3C). The expression of NOX2, caspase-3 and cleaved caspase-9 was significantly higher in DOX+Negative siRNA group than that in Negative siRNA and control groups. The expression of NOX2, caspase-3 and cleaved caspase-9 was significantly lower in DOX+NOX2 siRNA group than that in DOX+Negative siRNA group (Figure 3D, 3E). The expression of NOX4, caspase-3 and cleaved caspase-9 was significantly higher in DOX+Negative siRNA group than that in Negative siRNA and control groups. The expression of NOX4, caspase-3 and cleaved caspase-9 was significantly lower in DOX+NOX4 siRNA group than that in DOX+Negative siRNA group (Figure 3F, 3G). The expression of ROS was significantly higher in DOX+Negative siRNA group than that in Negative siRNA and control groups. The expression of ROS was significantly lower in DOX+NOX2 siRNA and DOX+NOX4 siRNA group than that in DOX+Negative siRNA group (Figure 3H, 3I). From the result, it was apparent that NOX2 siRNA and NOX4 siRNA can reduce the expressions of caspase-3, cleaved caspase-9 and the content of ROS in DOX-treated H9c2 cells.

### Overexpression of NOX2 and NOX4 enhanced ROS generation, caspase-3, and cleaved caspase-9 expressions in DOX-treated H9c2 cells

**Figure 4:**
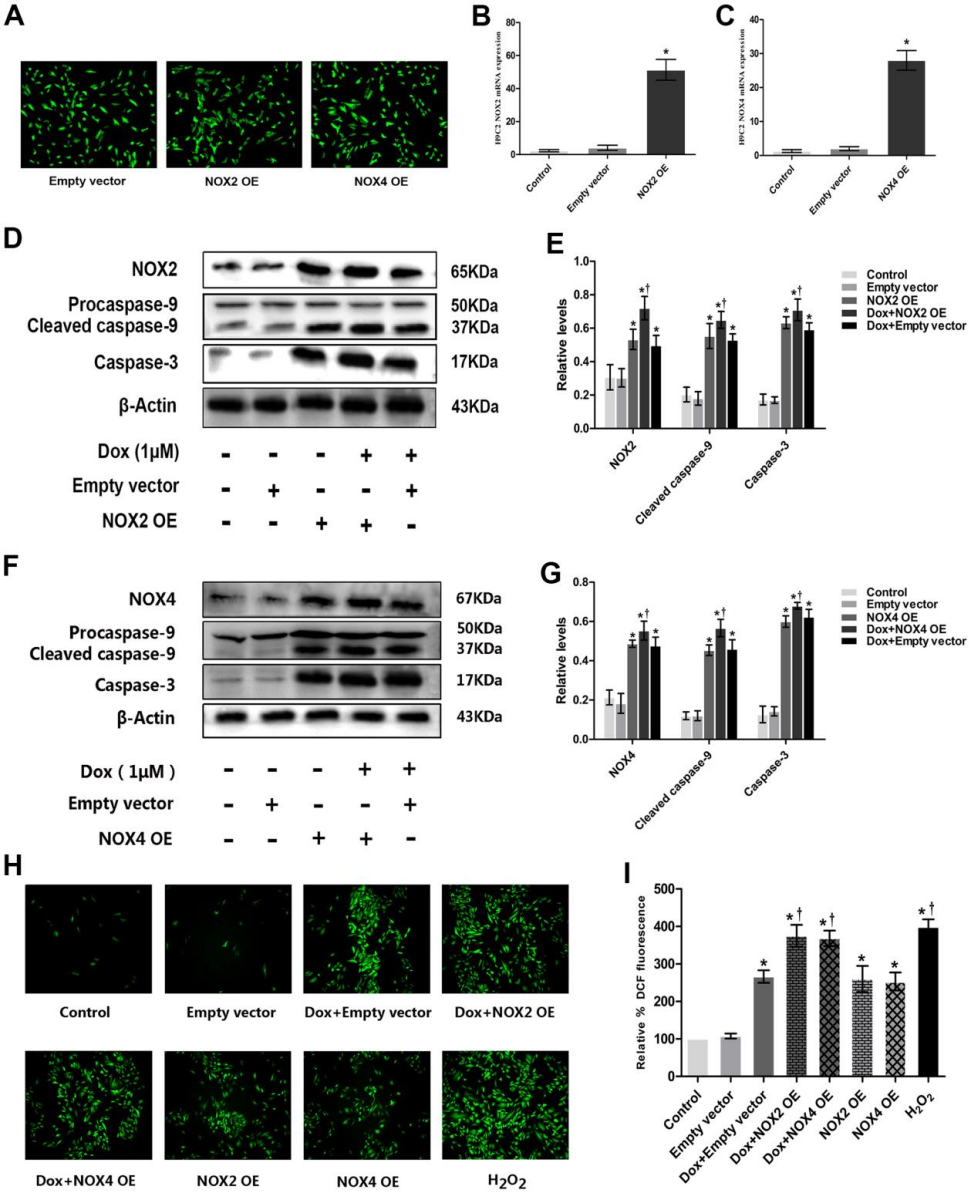
**Overexpression of NOX2 and NOX4 could increase DOX-induced ROS production and expression of apoptotic proteins in H9c2 cells.** (**A**) Fluorescence microscope observation of H9c2 cells after transfecting with Empty vector, NOX2 OE and NOX4 OE (×100). (**B**) The bar graph of NOX2 eukaryotic expression plasmid verified by RT-PCR. (**C**) The bar graph of NOX4 eukaryotic expression plasmid verified by RT-PCR. (**D**) Western blot analysis of NOX2 OE and DOX-treatment on NOX2, caspase-3, cleaved caspase-9 protein levels. (**E**) Densitometry analysis of the protein bands of NOX2, caspase-3, cleaved caspase-9 proteins. (**F**) Western blot analysis of NOX4 OE and DOX-treatment on NOX4, caspase-3, cleaved caspase-9 protein levels. (**G**) Densitometry analysis of the protein bands of NOX4, caspase-3, cleaved caspase-9 proteins. (**H**) Effect of NOX2 OE, NOX4 OE and DOX-treatment on ROS levels at 12 h time point in H9c2 cells. (**I**) Microscopic analysis of NOX2 OE, NOX4 OE and DOX-treatment on ROS levels by DCF Fluorescence. *P<0.05 vs. Control; †P<0.05 vs. Dox. Dox, doxorubicin; OE, overexpression.

H9c2 cells were transfected with Empty vector, NOX2 and NOX4 plasmids, respectively. The results show that the NOX plasmid has a good transfection effect (Figure 4A) and overexpression efficiency (Figure 4B, 4C). The expressions of NOX2, caspase-3 and cleaved caspase-9 were significantly higher in DOX+Empty vector group or NOX2 OE group than in Empty vector and control groups. The expressions of NOX2, caspase-3 and cleaved caspase-9 were significantly higher in DOX+NOX2 OE group than in DOX+Empty vector group (Figure 4D, 4E). The expressions of NOX4, caspase-3 and cleaved caspase-9 were significantly higher in DOX+Empty vector group or NOX4 OE group than in Empty vector and control groups. The expressions of NOX4, caspase-3 and cleaved caspase-9 were significantly higher in DOX+NOX4 OE group than that in DOX+Empty vector group (Figure 4F, 4G). The expression of ROS was significantly higher in DOX+Empty vector group than that in Empty vector and control groups. The expression of ROS was significantly higher in DOX+NOX2 OE and DOX+NOX4 OE groups compared to DOX+Empty vector group (Figure 4H, 4I). These results show that NOX2 OE and NOX4 OE can increase the expression of caspase-3, cleaved caspase-9 and the content of ROS in DOX-treated H9c2 cells.

### Effects of Val combined with MSCs on ROS generation, AT_1_R, NOX2, NOX4, caspase-3, and cleaved caspase-9 expressions in H9c2 cells

**Figure 5:**
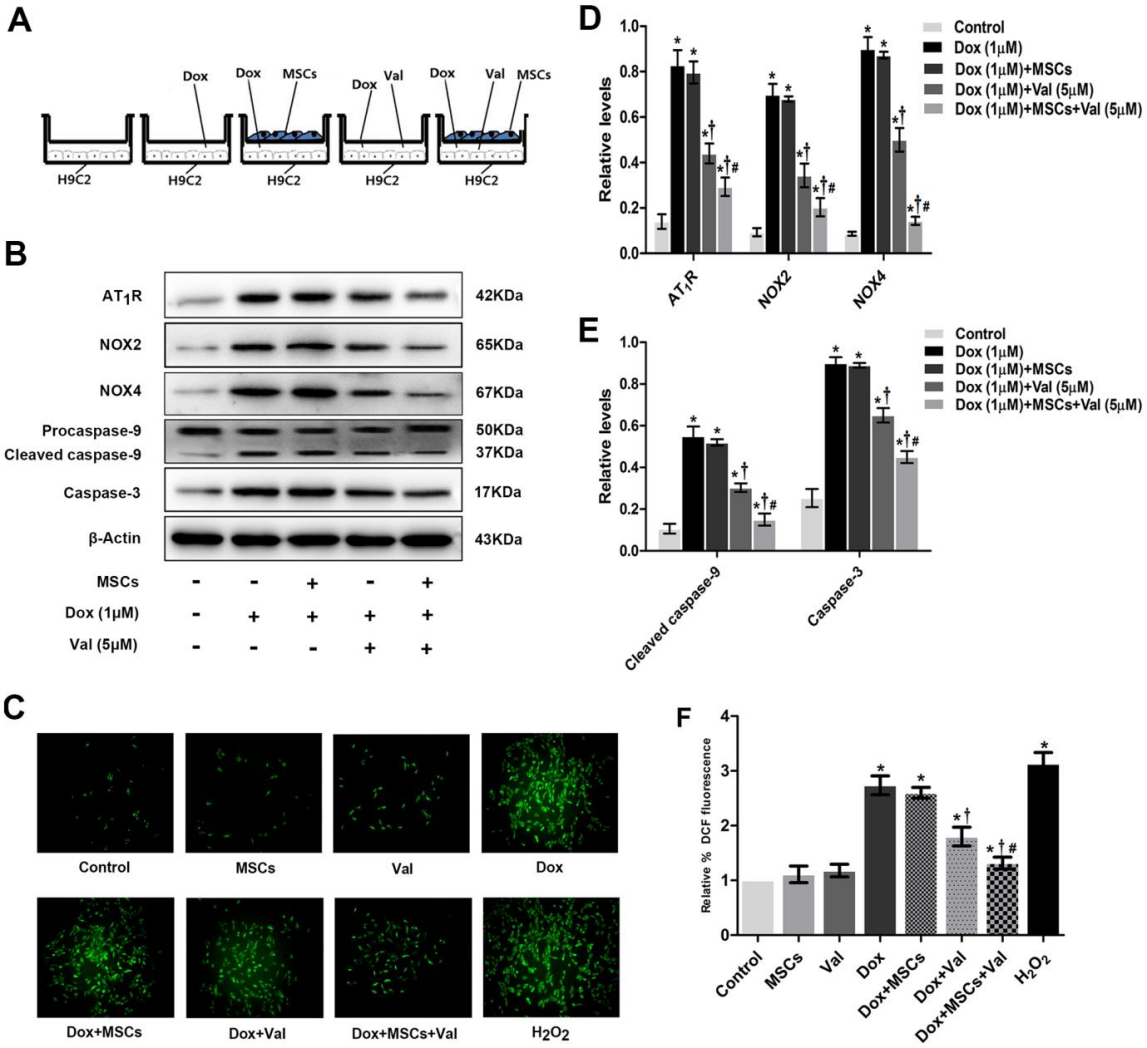
**MSCs combined with Val effects on ROS generation, AngII, NOX2, NOX4, caspase-3 and cleaved caspase-9 expression in H9c2 cells.** (**A**) Schematic diagram of co-culture of MSCs and H9c2 cells. (**B**) Western blot analysis of MSCs and Val-treatment on AT1R, NOX2, NOX4, caspase-3, cleaved caspase-9 proteins levels. (**C**) Effect of MSCs and Val-treatment on ROS levels at 12 h time point in H9c2 cells. (**D**, **E**) Densitometry analysis of the protein bands of AT1R, NOX2, NOX4, caspase-3, cleaved caspase-9 proteins. (**F**) Microscopic analysis of MSCs and Val-treatment on ROS levels by DCF Fluorescence. *P<0.05 vs. Control; †P<0.05 vs. Dox; #P<0.05 vs. Dox+Val, Dox+MSCs. Dox, doxorubicin; Val, valsartan; MSCs, mesenchymal stem cells.

To verify if there is a synergic protective effect of MSC with Val, we co-cultured cells with both MSC and Val and used the Transwell system to prevent direct exposure of MSCs to H9c2 cells (Figure 5A). The expressions of AT_1_R, NOX2, NOX4, caspase-3 and cleaved caspase-9 were significantly higher in DOX group than in control group. The expressions of AT_1_R, NOX2, NOX4, caspase-3 and cleaved caspase-9 were similar between DOX+MSCs group and DOX group. However, the expressions of AT_1_R, NOX2, NOX4, caspase-3 and cleaved caspase-9 were significantly lower in DOX+MSCs+Val group than in DOX+MSCs and DOX+Val groups (Figure 5B, 5D, 5E). The generation of ROS was significantly higher in DOX group than in control group. The generation of ROS was similar between DOX+MSCs group and DOX group. But the generation of ROS was significantly lower in DOX+MSCs+Val group than in DOX+MSCs and DOX+Val groups (Figure 5C, 5F). From the result, it was apparent that MSCs therapy alone could not effectively reduce the expressions of AT_1_R, NOX2, NOX4, caspase-3 and cleaved caspase-9 proteins and the generation of ROS in H9c2 cells treated with DOX. Taken together, MSCs enhanced the protective effects of Val on alleviating the DOX-induced cytotoxicity in H9c2 cells.

### Impact of Val, NOX (knockdown and overexpression), MSCs on MAPK signaling pathway in DOX-treated H9c2 cells

**Figure 6:**
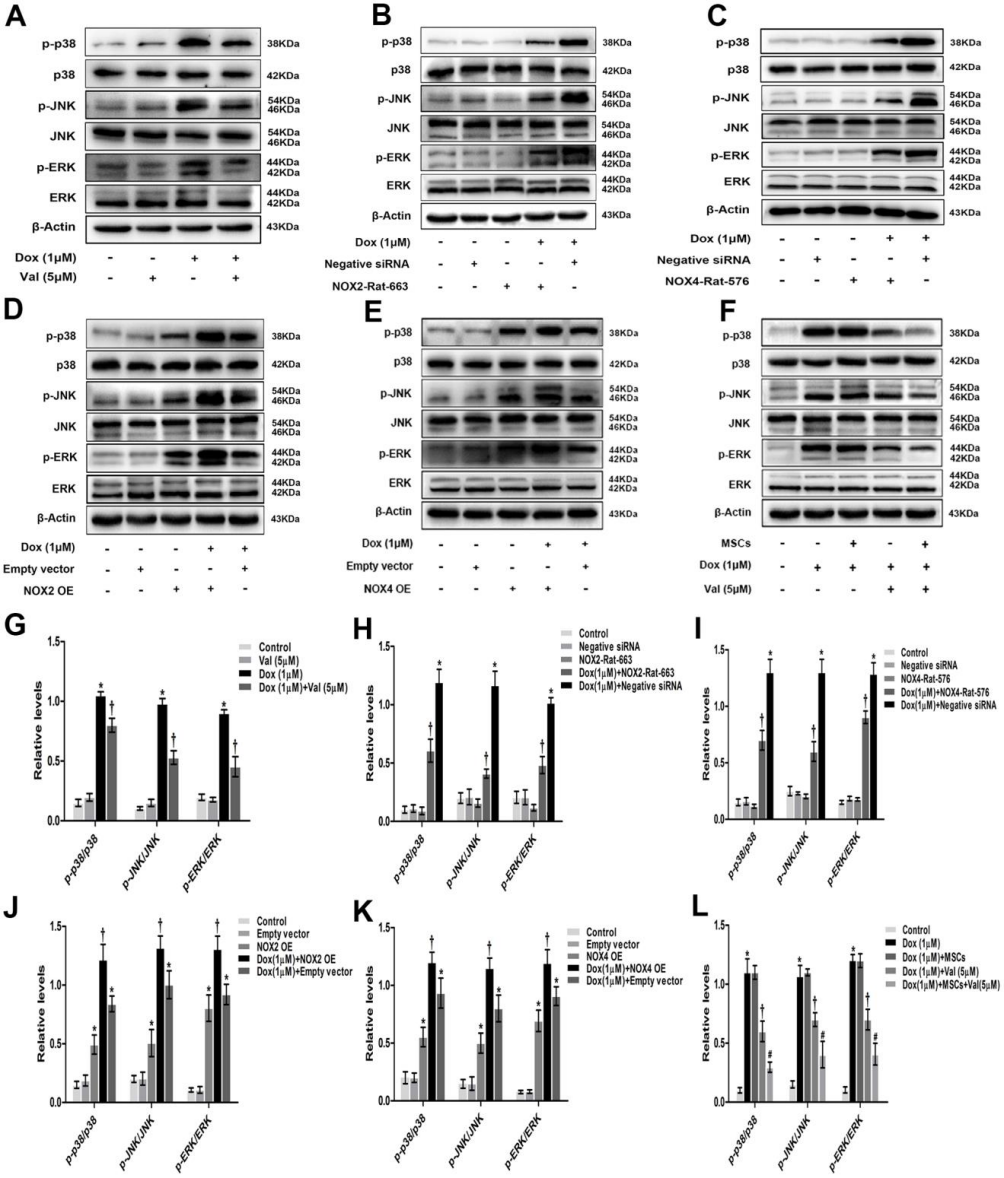
**Val, NOX (knockdown and overexpression), MSCs effects on MAPK signaling pathway in H9c2 cells.** (**A**) Western blot analysis of DOX and Val-treatment on p-p38, p-JNK, p-ERK proteins levels. (**B**, **C**) Western blot analysis of NOX2 siRNA and NOX4 siRNA treatment on p-p38, p-JNK, p-ERK proteins levels. (**D**, **E**) Western blot analysis of NOX2 plasmid and NOX4 plasmid treatment on p-p38, p-JNK, p-ERK proteins levels. (**F**) Western blot analysis of MSCs and Val-treatment on p-p38, p-JNK, p-ERK proteins levels. (**G**) Densitometry analysis of the protein bands of p-p38, p-JNK, p-ERK after Val and DOX treatment. (**H**, **I**) Densitometry analysis of the protein bands of p-p38, p-JNK, p-ERK after NOX2 siRNA and NOX4 siRNA treatment. (**J**, **K**) Densitometry analysis of the protein bands of p-p38, p-JNK, p-ERK after NOX2 plasmid and NOX4 plasmid treatment. (**L**) Densitometry analysis of the protein bands of p-p38, p-JNK, p-ERK after MSCs and Val treatment. *P<0.05 vs. Control, Negative siRNA or Empty vector; †P<0.05 vs. Dox or DOX+Negative siRNA or DOX+Empty vector; #P<0.05 vs. DOX+Val or DOX+MSCs. DOX, doxorubicin; Val, valsartan; MSCs, mesenchymal stem cells.

p-p38, p-JNK, p-ERK protein expressions in DOX-treated H9c2 cells was evaluated in the presence of Val, MSCs, NOX siRNA or plasmid. The expressions of p-p38, p-JNK, p-ERK were significantly higher in DOX group than in control group. The expressions of p-p38, p-JNK, p-ERK were significantly lower in DOX+Val group than that in DOX group (Figure 6A, 6G). The expressions of p-p38, p-JNK, p-ERK were significantly higher in DOX+Negative siRNA group than in Negative siRNA group. The expressions of p-p38, p-JNK, p-ERK were significantly lower in DOX+NOX2 siRNA and DOX+NOX4 siRNA groups than in DOX+Negative siRNA group (Figure 6B, 6C, 6H, 6I). The expressions of p-p38, p-JNK, p-ERK were significantly higher in DOX+Empty vector group or NOX2 OE group or NOX4 OE group than in Empty vector group. The expressions of p-p38, p-JNK, p-ERK were significantly higher in DOX+NOX2 OE and DOX+NOX4 OE groups than in DOX+Empty vector group (Figure 6D, 6E, 6J, 6K). The expressions of p-p38, p-JNK, p-ERK were similar between DOX+MSCs group and DOX group. However, the expressions of p-p38, p-JNK, p-ERK were significantly lower in DOX+MSCs+Val group than in DOX+MSCs and DOX+Val groups (Figure 6F, 6L). From the result, it was apparent that DOX could activate the MAPK signaling pathway, which was characterized by increased protein expressions of p-p38, p-JNK, p-ERK. NOX2 and NOX4 were involved in the activation of MAPK signaling pathway induced by DOX. Val could reduce the expression of p-p38, p-JNK, p-ERK proteins, and MSCs alone could not reduce these proteins. However, MSCs enhanced the protective effects of Val in DOX-treated H9c2 cells.

## DISCUSSION

**Figure 7:**
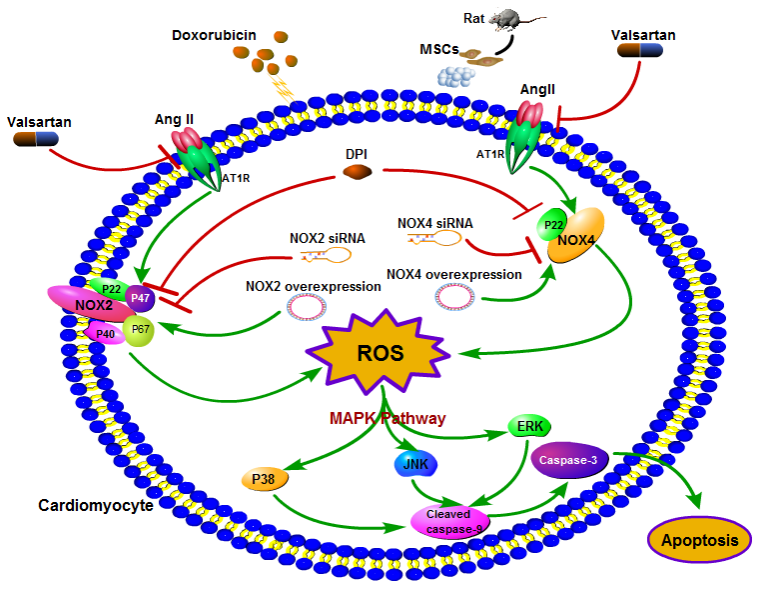
Schematic diagram of the effect of Val combined with MSCs on DOX-induced apoptosis in H9c2 cardiomyocytes.

DOX-induced cardiotoxicity could be presented in forms of dilated cardiomyopathy and congestive heart failure [21]. Previous study found that DOX could increase RAAS activity and increase the content of ROS in the rat model of DOX-induced dilated cardiomyopathy (DCM) [22]. The present study indicates that AT1R/NOX/ROS/MAPK signaling pathway is involved in DOX-induced cardiotoxicity and confirmed the previous finding that Val treatment significantly attenuated DOX-induced cardiotoxicity, without affecting the anti-tumor effect of DOX. The novel finding of the present study is that MSCs enhanced the protective effects of Val in alleviating the toxic effects of DOX in H9c2 cells. To our best knowledge, this is the first *in vitro* report describing the synergic protective effects of Val and MSCs and their impact on the AT1R/NOX/ROS/MAPK signaling in H9c2 cells, The Schematic diagram of the effect of Val combined with MSCs on DOX-induced apoptosis in H9c2 cardiomyocytes mentioned in (Figure 7).

### AngII/NOX/ROS/MAPK signaling mediated DOX-induced cytotoxicity

The present study evidenced that AT_1_R, NOX2, NOX4 as well as apoptosis related caspase-3 and cleaved caspase-9 protein expression are significantly upregulated post DOX stimulation in H9c2 cells (Figure 2A, 2B), which might be responsible for the enhanced apoptosis and ROS formation (Figure 1). As expected, these changes could be reversed by cotreatment with Val (Figure 2A, 2B). To elucidate the causal role of NOX2 and NOX4 signaling, we observed the impact of DPI, a NADPH inhibitor with known effects on inhibiting NOX2 and NOX4, in the DOX-treated H9c2 cells. It was shown that DPI significantly downregulated the protein expression of caspase-3 and cleaved caspase-9, reduced apoptosis and ROS formation (Figure 2C–2F). These effects suggest the close link between NOX2 and NOX4 upregulation and DOX-induced cytotoxicity. Similarly, experiments with NOX2 siRNA and NOX4 siRNA in DOX-treated H9c2 cells reveal that knockdown of NOX2 and NOX4 also induce downregulated caspase-3 and cleaved caspase-9 expression, followed by reduced ROS formation (Figure 3). Meanwhile, overexpression of NOX2 and NOX4 enhanced DOX-induced ROS formation and apoptosis, as well as caspase-3 and cleaved caspase-9 upregulation (Figure 4), indicating a causal role of NOX2 and NOX4 signaling on DOX-induced cytotoxicity. Moreover, we found that changes on protein expressions of p-p38, p-JNK and p-ERK coincidence with MAPK signaling activation-induced apoptosis (Figure 6). Downregulated MAPK signaling was observed post Val intervention in our study. Previous study showed that MAPK signaling pathway was associated with apoptosis [23]. We thus speculate inhibiting MAPK pathway might be one of the working mechanisms of Val in this model. However, more evidence is needed to show MAPK signaling is a mediator for Val's protective effect upon DOX treatment. Future studies are warranted to observe if the beneficial effects of Val could be reversed or not in the co-presence of MAPK pathway agonist. Thus, our study suggest that the AngII/NOX/ROS/MAPK pathways play a major role in DOX-induced cardiotoxicity.

### The potential mechanism of the synergic effects of MSCs on Val-mediated protective effects in DOX-stimulated H9c2 cells

Previous study by our group found that repeated intravenous infusion of MSCs could improve the cardiac function of rats with DOX-induced dilated cardiomyopathy and reduce myocardial fibrosis [12]. The study showed that MSCs could reduce the mRNA expression of AT1, CYP11B2, TGF-β, collagen I and collagen III in rat myocardium. The decreased expression of AT1 and CYP11B2 might indicate that MSCs inhibited the RAAS system in DOX-treated rats. Val, as a receptor antagonist of Ang II, which jointly inhibited the Ang II type 1 (AT1) receptor, thereby reducing the activity of the RAAS [13]. Due to the synchronized effect of MSCs and Val on RAAS, the study protocol with MSCs and Val might consequently reduce the activation of oxidative stress and cell apoptosis, thereby alleviate the myocardial cytotoxicity induced by DOX. We thus tested the hypothesis that MSCs might enhance the protective effects of Val on attenuating DOX-induced cytotoxicity in H9c2 cells (Figure 5). Although MSCs alone, did not affect the MAPK signaling in this model, MSCs+Val treatment induced significant downregulation on protein expressions of MAPK pathway (p-p38, p-JNK, p-ERK) as compared to that of Val alone in DOX-stimulated H9c2 cells (Figure 6). This indicates that the observed synergic beneficial effects in this cell model might be mediated via modulating the MAPK pathway. Future studies are warranted to validate the mechanisms both *in vitro* and *in vivo* as well as in clinical studies.

### Other potential mechanisms of the synergic effects of MSCs+Val on DOX-induced toxicity

Previous studies demonstrated that ATIIR1and/or ATIIR2 signaling are crucially involved in the MSCs differentiation into endothelia cells and hematopoietic cells [24]. Previous experiments also elucidated the role of MSCs on non-ischemic dilated cardiomyopathy through improving the endothelial function [25–27]. We thus speculated that the interaction of MSCs and Val might be a useful strategy in that Val could facilitate the differentiation of MSCs into endothelial and hematopoietic cells, thereby alleviating DOX-induced cytotoxicity.

Enhanced proinflammatory cytokines, including TNF-α, belong to the pathogenesis of DOX-induced cytotoxicity [28]. Studies show that hMSC could reduce the release of multiple proinflammatory cytokines including TNF-α [29]. In fact, it was shown that ARB could also reduce TNF-α levels [30]. It is thus possible that the synergic effect of MSCs with Val in this *in vitro* model might be mediated by modulating the TNF-α and future studies are needed to validate this hypothesis.

### Impact of Val on the anti-tumor effect of DOX

To exclude the negative impact of Val on the antitumor effect of DOX, we explored the impact of Val on MDA-MB-231cells and A549 cells, and results showed that cell viability was similar between DOX treated MDA-MB-231 cells or A549 cells and DOX+Val treated MDA-MB-231 cells or A549 cells, so that Val could be used to attenuate DOX-induced cytotoxicity without affecting the antitumor effect of DOX.

## CONCLUSIONS

In summary, AT_1_R-NOX-ROS-MAPK signaling pathway is involved in the DOX-induced cardiotoxicity. Val treatment could attenuate DOX-induced cytotoxicity through modulating this pathway without affecting the anti-tumor effect of DOX. MSCs could enhance the beneficial effects of Val against DOX-induced cytotoxic effects through modulating AT_1_R/NOX/ROS/MAPK signaling pathway in H9c2 cells.
